# Using a multinomial tree model for detecting mixtures in perceptual detection

**DOI:** 10.3389/fpsyg.2014.00641

**Published:** 2014-06-27

**Authors:** Richard A. Chechile

**Affiliations:** Psychology Department, Tufts UniversityMedford, MA, USA

**Keywords:** signal detection theory, multinomial processing tree models, perceptual learning, mixture detection, shrinkage estimators

## Abstract

In the area of memory research there have been two rival approaches for memory measurement—signal detection theory (SDT) and multinomial processing trees (MPT). Both approaches provide measures for the quality of the memory representation, and both approaches provide for corrections for response bias. In recent years there has been a strong case advanced for the MPT approach because of the finding of stochastic mixtures on both target-present and target-absent tests. In this paper a case is made that perceptual detection, like memory recognition, involves a mixture of processes that are readily represented as a MPT model. The Chechile (2004) 6P memory measurement model is modified in order to apply to the case of perceptual detection. This new MPT model is called the Perceptual Detection (PD) model. The properties of the PD model are developed, and the model is applied to some existing data of a radiologist examining CT scans. The PD model brings out novel features that were absent from a standard SDT analysis. Also the topic of optimal parameter estimation on an individual-observer basis is explored with Monte Carlo simulations. These simulations reveal that the mean of the Bayesian posterior distribution is a more accurate estimator than the corresponding maximum likelihood estimator (MLE). Monte Carlo simulations also indicate that model estimates based on only the data from an individual observer can be improved upon (in the sense of being more accurate) by an adjustment that takes into account the parameter estimate based on the data pooled across all the observers. The adjustment of the estimate for an individual is discussed as an analogous statistical effect to the improvement over the individual MLE demonstrated by the James–Stein shrinkage estimator in the case of the multiple-group normal model.

## 1. Introduction

The title of this special issue implies two very different questions. The first question is: how should perceptual decision-making be modeled? The second question is: how should individual differences be estimated? This paper addresses both of these questions from a perspective that has been informed by research in the area of model-based memory measurement. The recommendations from this perspective result in some novel techniques for examining perceptual detection data.

Signal detection theory (SDT) is the classic method for measuring the perceived strength of a stimulus (Tanner and Swets, 1954; Green and Swets, 1966). The original applications of SDT typically dealt with cases of detecting the presence of a slight intensity increase on a single sensory dimension such as the loudness of white noise or an increase in the brightness of a color patch. The data from these studies are multinomial frequencies that are used to estimate either a signal sensitivity measure (*d*′) associated with the separation between two presumed distributions on a psychological strength continuum, or a non-parametric measure such as *A*′ associated with the area under the receiver-operator characteristic (ROC) curve. For such applications there has been a general consensus that SDT is valid, accurate and useful. SDT has also been extended to the case of multiple dimensions (e.g., Ashby and Townsend, 1986).

Egan (1958) first noted that the target-present versus target-absent test trials used in a yes/no recognition memory study correspond to the signal-present versus signal-absent tests used in a sensory-based signal detection task. It therefore followed that SDT provided a method for measuring memory strength. In fact Macmillan and Creelman (2005) observed that contemporary applications of SDT in the memory area outnumbered the psychophysical applications. Malmberg (2008) and Yonelinas (2002) provide extensive reviews of recognition memory from the perspective of strength-based SDT models. Yet despite the widespread use of the SDT approach toward recognition memory measurement, there also has been substantial criticism of this approach (Chechile, 1978, 2013; Bröder and Schütz, 2009; Kellen et al., 2013). These critics argue instead for the use of multinomial process tree (MPT) models for a variety of reasons. MPT models have a number of desirable statistical properties and can result in measurements of important latent cognitive processes. For example Chechile and Meyer (1976) first used MPT models for recognition memory data as well as recall data in order to obtain separate probability measures for trace storage and for the retrieval of stored traces, because forgetting was more suitably described in terms of either storage failures or retrieval failures rather than simply a change in “memory strength.” The implicit-explicit separation (IES) model is another example of a MPT model rather than a SDT model for memory (Chechile et al., 2012). With the IES model separate probability measures are estimated for explicit storage, implicit storage, fractional storage and non-storage. In these examples, the MPT modeler deliberately prefers to measure cognitive processes other than a SDT strength measure. See Erdfelder et al. (2009) and Batchelder and Riefer (1999) for additional examples of MPT models in psychology.

MPT models are mixture models because with this approach it is assumed that there are possibly different knowledge states that have differential consequence for behavior. For example, sometimes there is enough information stored in memory that the individual can reproduce the target event entirely, provided that the information is accessible at the time of test. But for other tests, the requisite information is either incomplete or totally missing. In the Chechile (2004) 6P model there are separate tree pathways for these two different knowledge states. The overall proportion of traces that are sufficiently stored is defined as the storage probability θ_*S*_. The θ_*S*_ parameter is thus a mixture component. Similarly the other parameters in the 6P model are also probabilities and can be regarded as conditional mixture probabilities. Chechile (2013) provided strong evidence for the necessity of considering mixtures for both target-present memory tests as well as for target-absent tests. Evidence was also provided that mixtures are difficult to detect, i.e., data can be generated where a mixture is present but where conventional density plots or quantile–quantile plots fail to detect the mixture. In contrast MPT models are an excellent method for detecting mixtures. Moreover, the absence of a mixture is a special case of a MPT model where the tree paths have probabilities of either 0 or 1^1^.

While there is an ongoing debate about SDT and MPT models in the memory literature, there has not been a corresponding contemporary debate in perceptual psychology about the relative merits of SDT and MPT approaches. Yet the possibility of stochastic mixtures is quite plausible for perceptual detection studies, so there are reasons for considering MPT models for perceptual detection.

One rationale for suspecting that there are mixtures comes from the Stevens (1957, 1961) distinction between prothetic and metathetic continua. Stevens (1961); Stevens (p. 41) illustrated a prothetic dimension with loudness and distinguished it from pitch, which is regarded as a metathetic continuum:

… it is interesting that some of the better known prothetic continua seem to be mediated by an *additive* mechanism at the physiological level, whereas the metathetic continua appear to involve *substitutive* processes at the physiological level. Thus we experience a change in loudness when excitation is added to excitation already present on the basilar membrane, but we note a change in pitch when new excitation is substituted for excitation that has been removed, i.e., the pattern of excitation is displaced

The Stevens distinction stresses the difference between changes in intensity on a single dimension and changes in qualities. A homogeneous process (as opposed to a mixture) is more likely when dealing with a prothetic continuum; although DeCarlo (2002, 2007) has pointed out that trial-by-trial shifts in attention or phasic alertness can produce a stochastic mixture even in a perceptual detection task on a single dimension. However, if the stimuli are complex and possess qualitative features, then stochastic mixtures are even more likely. Consider, for example, a sonar operator attempting to detect any enemy threats. The operator might detect a clear auditory pattern that is a prototypical signal of a particular class of an enemy submarine. With training and experience the sonar operator can be highly skilled in detecting the complex set of features that are associated with an enemy threat; after all perceptual learning is a well established fact (Kellman, 2002). From this framework, the operator might confidently detect a target, not because of a greater strength or intensity, but because the metathetic pattern exhibited by the stimulus is linked through training to a particular type of target. Yet there might be other cases when a threat is present, but the sonar signal is too poorly defined to be identified as a threat. The operator has to guess in these cases. Hence, from this perspective targets stimuli can be considered a mixture of occasions where the target is confidently and correctly identified and other occasion where the operator guesses. A mixture is also possible over all the target-absent cases. For example, a sonar operator might decide that the stimulus is something other than an enemy threat (e.g., a party boat, or a whale), but for other target-absent events the signal might be too poorly defined for the sonar operator to confidently identify. In this paper, a variation of a MPT model will be advanced for perceptual-detection applications in order to capture the possibility that there are mixtures reflected in the data.

The second focus for this paper concerns the relative accuracy of various statistical procedures for modeling individual differences in terms of the key parameters of a perceptual detection MPT model. There is a widespread belief that the maximum likelihood estimates (MLE) of model parameters, done on an individual basis, is the optional method for obtaining estimates of individual differences. This belief is mistaken; there is now considerable evidence that the MLE can be non-optimal and biased for a number of important practical cases. Even in the case of the Gaussian model with more than two conditions, the MLE estimates are known to be biased and “inadmissible” due to the Stein paradox (Stein, 1956; James and Stein, 1961; Efron and Morris, 1977). These insights have led to empirical Bayes, James–Stein estimators, and other shrinkage estimators as improvements to the MLE (Efron and Morris, 1973; Gruber, 1998). Moreover, based on Monte Carlo simulations of multinomial data, Chechile (2009) found that the averaging of individual parameter estimates resulted in greater error than pooling the multinomial data across individuals and fitting the MPT model once. This finding foreshadows a relatively surprising result that is similar to the James–Stein shrinkage estimate for individual model parameter estimates.

## 2. The perceptual-detection (PD) MPT model

### 2.1. Data structure and tree model

**Figure 1:**
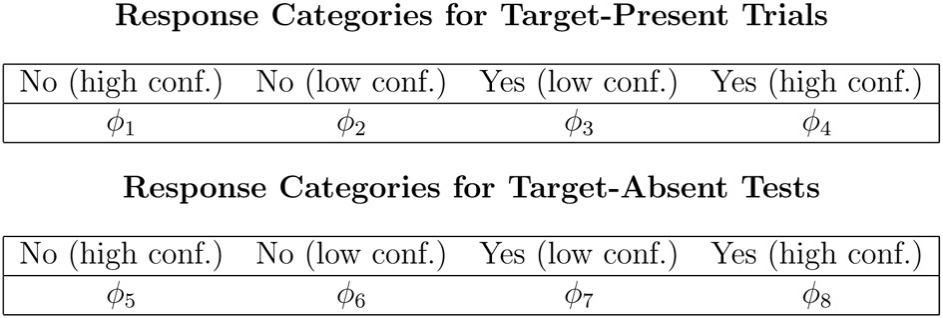
**Data categories and population proportions for the PD model**.

**Figure 2:**
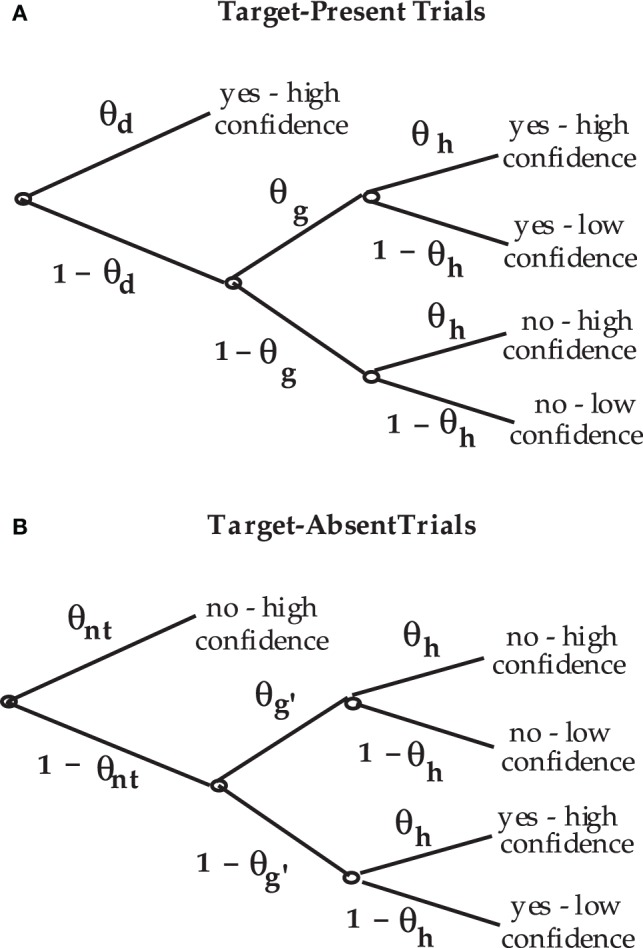
**Process tree for the PD model for (A) target-present test trials and (B) target-absent test trials**.

The Perceptual-Detection (PD) model is essentially the Chechile (2004) 6P model for old/new recognition test trials. The 6P model for storage and retrieval components of memory also has a recall test that is not a part of the perceptual-detection task. The data categories for target-present and target-absent trials as well as the notation for the corresponding population proportions for each response category are shown in Figure 1. The PD tree is displayed in Figure 2. The MPT model has five parameters; the 6P model had an additional retrieval parameter that is not relevant for perceptual detection. The subscripts for the five parameters have been labeled differently in order to better match the perceptual detection context. The θ_*d*_ parameter is the proportion of target-present tests when the operator clearly and confidently detects the target stimulus; this parameter corresponds to the sufficient storage parameter θ_*S*_ in the 6P model. The θ_*nt*_ parameter is the proportion of the target-absent trials when the operator can confidently identify a stimulus that is different than the target; this parameter corresponds to the knowledge-based foil rejection parameter θ_*k*_ in the 6P model.

The θ_*d*_ and 1 − θ_*d*_ parameters are mixing rates for target-present trials. When the target is not clearly detected, the observer can still decide that the stimulus is a target (with conditional probability θ_*g*_) by a secondary process that is simply labeled as a guessing process. Similarly on target-absent tests, the operator (with probability 1 − θ_*nt*_) fails to confidently identify a non-target but can still guess (with probability θ_*g*′_) that the stimulus is more likely a non-target than a target. The two guessing parameters in the PD model are the same as the guessing parameters in the 6P model. Finally the θ_*h*_ parameter is a “nuisance” parameter because it is a conditional probability that is only important as a correction for overly confident guessing. This parameter corresponds to the θ_1_ parameter in the 6P model.

### 2.2. Parameter estimation and a radiology example

A great deal is known about the 6P model, and this information directly transfers to the PD model. For example, Chechile (2004) formally proved that the model is likelihood identifiable, i.e., each configuration of the model parameters results in a unique multinomial likelihood function^2^. Chechile (2004) also showed how the maximum likelihood estimates (MLE) are obtained for the model parameters. In that same paper, an exact Bayesian method for drawing random vectors of values from the posterior distribution was described; the method is called the population parameter mapping (PPM) method (see Chechile, 1998, 2010a). With the PPM method there is a full probability distribution for each model parameter, and there is a probability for the coherence of the model itself. Software also exists for obtaining random vectors from an approximate Bayesian posterior distribution by means of a Markov chain Monte Carlo (MCMC) sampling system^3^. For both the PPM method and the MCMC method, there is a point estimate for each parameter along with a Bayesian posterior probability distribution^4^. The PPM method has several advantages over the MCMC method. First, it does not require a “burn in” period. Second, the posterior distribution is exact as opposed to asymptotically exact. Third, the samples from the posterior distribution are not autocorrelated. Fourth, the PPM method has a probability for the coherence of the model itself.

As an example of parameter estimation for the PD model, let us consider the actual case of the detection characteristics of a single radiologist who was assessing 109 CT scans in order to detect abnormal versus normal scans. Hanley and McNeil (1982) provided the frequencies in four response categories. The categories were labeled as (1) “definitely normal,” (2) “probably normal,” (3) “probably abnormal,” and (4) “definitely abnormal.” There were a total of 58 patients who were later determined to be normal, and 51 patients who were determined later to have an abnormality. The frequencies in these four respective categories for the normals (target-absent) are (33, 9, 14, 2)^5^. The corresponding frequencies for the abnormals (target-present) are (3, 3, 12, 33)^6^. The PPM, MCMC, and MLE point estimates for each parameter in the PD model are displayed in Table 1.

**Table 1:** **PPM, MCMC, and MLE values for the PD model parameters from 109 CT scans by one radiologist reported in the Hanley and McNeil (1982) study**.

**Parameter**	**PPM**	**MCMC**	**MLE**
θ_*d*_	0.552	0.555	0.578
θ_*nt*_	0.496	0.507	0.523
θ_*g*_	0.734	0.711	0.721
θ_*g*′_	0.405	0.438	0.421
θ_*h*_	0.250	0.259	0.227

The PD model point estimates fit the multinomial frequencies very well as indicated by a non-significant goodness-of-fit difference between the observed and predicted frequencies, i.e., *G*^2^(1) = 0.262. In addition to the point estimates, the two Bayesian methods have a posterior probability distribution for each model parameter, and these distributions provide a method for testing some important questions about the radiologist. One of the central ideas in the PD model is the concept that there is a mixture of states for both target-present cases (abnormals) and for target-absent cases (normals). From the posterior distribution of the θ_*d*_ parameter, it can be stated that the probability exceeds 0.95 that the θ_*d*_ parameter is at least 0.39, i.e., *P*(θ_*d*_ > 0.39) > 0.95. Similarly the posterior distribution for the θ_*nt*_ parameter results in the high probability statement that θ_*nt*_ is at least 0.37, i.e., *P*(θ_*nt*_ > 0.37) >0.95.

Using a standard SDT model analysis of the radiological data results in an estimate of *d*′ = 2.332 and a ratio of the standard deviations between the signal and noise conditions of $\frac{\sigma_{S}}{\sigma_{N}}=1.409$. This model also fits the data well as indicated by a non-significant difference between the observed and expected frequencies, *G*^2^(1) = 0.220. However, the SDT model does not posit that there are mixtures, so the finding that the θ_*d*_ and θ_*nt*_ parameters are reliably different than zero demonstrates that the conventional signal detection model is missing an important feature exhibited by the radiologist. If there were an absence of mixtures, then the PD model would have estimated the θ_*d*_ and θ_*nt*_ parameters as approximately 0.

For MPT models, the mean of the Bayesian posterior distribution for a parameter is usually a different value than the MLE. Chechile (2004) conducted a series of Monte Carlo simulations to see which of these estimates is more accurate for the 6P model; these simulations directly apply to the PD model. For each Monte Carlo run, a random configuration of the model parameters was selected. These parameter values became the true values that are compared later to the estimated values. Also based on the true values, there is a corresponding set of true multinomial cell proportions, i.e., the ϕ_*i*_ values in Figure 1. From the multinomial likelihood distributions, *n* random “observations” were drawn for the target-present frequencies and another *n* random observations were drawn for the target-absent frequencies^7^. Using the cell frequencies, the PPM and MLE parameter estimates are computed. For each estimate there is thus an error score based on the absolute value difference between the estimated value and the true value for that particular Monte Carlo run. For each sample size there was a total of 10,000 Monte Carlo runs. The mean absolute value across the 10,000 runs for PPM and MLE methods are denoted respectively as MAE(ppm) and MAE(mle). The standard deviation of the absolute value errors was also found for both estimation methods. Representative results from these Monte Carlo simulations are shown in Table 2 for the θ_*d*_ parameter.

**Table 2:** **The mean absolute value error (MAE) for the θ_*d*_ parameter for both the PPM and MLE methods**.

***n***	**MAE(ppm)**	**MAE(mle)**	**SDE(ppm)**	**SDE(mle)**
10	0.129	0.198	0.090	0.174
20	0.102	0.143	0.076	0.135
30	0.090	0.124	0.070	0.123
40	0.082	0.112	0.064	0.115
50	0.075	0.099	0.061	0.104
100	0.059	0.071	0.050	0.074
300	0.039	0.043	0.035	0.047
600	0.029	0.030	0.027	0.031
1000	0.023	0.023	0.022	0.025

*Also shown are the standard deviations of the errors (SDE). Each entry is based on 10,000 Monte Carlo runs from Chechile (2004)*.

The Bayesian PPM estimates are more accurate for all the sample sizes. Although the MLE and PPM errors are approaching each other, the rate of approach is relatively slow. Notice that even for the case of *n* = 1000, there is still a smaller standard deviation of the errors for the PPM estimates. The greater accuracy for the Bayesian PPM estimates has been also demonstrated for other MPT models (Chechile, 2009, 2010a).

### 2.3. Interpreting the guessing parameters

The θ_*g*_ and θ_*g*′_ parameters have actually been used in memory applications since the original storage-retrieval separation paper by Chechile and Meyer (1976). In the memory context it was hypothesized that the guessing parameters involve a mixture of processes that include the possibility of partial storage as well as response bias factors. For memory applications, these parameters are both typically greater than $\frac{1}{2}$, (viz. Chechile and Ehrensbeck, 1983; Chechile and Meyer, 1976; Chechile, 1987, 2004, 2010b; Chechile and Roder, 1998). If the guessing parameters were strictly response bias, then both parameters should not exceed $\frac{1}{2}$, but if there is sometimes partial storage, then that information can be helpful and result in the two guessing parameters exceeding $\frac{1}{2}$. Although the possibility of partial storage was likely, it was not possible to estimate fractional storage with only the yes/no recognition data along with confidence ratings. Later Chechile and Soraci (1999) and Chechile et al. (2012) used different test protocols that enabled the measurement of partial storage. These other MPT models did find evidence for partial storage on some test trials; consequently, the finding of both guessing parameters being greater than $\frac{1}{2}$ is a reasonable outcome.

For the PD model, there is a counterpart to the educated guessing based on partial storage. For the perceptual detection task, there might be occasions where a stimulus is judged more likely a target than not but the quality of the perception is not good enough to constitute a confident classification. On other occasions, the stimulus might be judged more likely a particular “non-target” than a target, but again because the stimulus quality is degraded, the observer is uncertain. For both cases the stimulus is not in a clear detection state, but nonetheless, the person is still able to make informed decisions above a random guessing level.

An interesting special case is when the guessing in both target-present and target-absent conditions are purely response bias, i.e., when θ_*g*_ = 1 −θ_*g*′_. However, if there is something like the partial storage found for some memory studies, then the stimulus is more likely to yield a yes response in the target-present condition than in the target-absent condition. Note that the radiologist measured with the PD model exhibited guessing better than pure response bias because θ_*g*_ = 0.734 > 1 − θ_*g*′_ = 0.595. These results are consistent with the interpretation that the radiologist was relatively conservative because the doctor guessed that the patient had an abnormality at a rate of 0.595 for the subset of difficult scans from healthy patients. Nonetheless for the subset of difficult scans from patients with an abnormality, the rate for deciding on the abnormal categorization increased to 0.734. Consequently on these more challenging CT scans the physician did have some differential tendency to use the abnormal classification when in fact the CT scan came from a patient with an abnormality.

### 2.4. Properties of the ROC for the PD model

The Receiver Operator Characteristic (ROC) in SDT is a curved plot of the hit rate versus the false alarm rate. In standard SDT, any point on the ROC is a possible operating point depending on the decision criterion used by the subject. Hence in standard SDT, the ROC is an iso-sensitivity curve. In standard SDT, the points (0, 0) and (1, 1) are on the ROC curve; these points are the extrema. If the subject had no ability to detect the target, and the data are identical in the target-absent and target-present conditions, then the ROC would be the line of slope 1 connecting the extrema. If there is some greater tendency to detect the target in the target-present condition, then in standard SDT the ROC is a smooth curve in the region of the unit square where *y* ≥ *x*.

**Figure 3:**
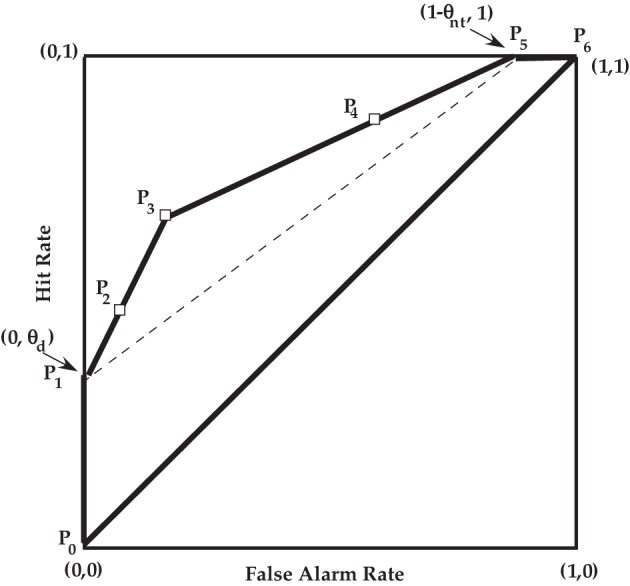
**ROC for the PD model**. See Table 3 for a description of the points *P*_*i*_, *i* = 0, …, 6. The open squares are the three theoretical “operating” points, and the extrema points are *P*_1_ = (0, θ_*d*_) and *P*_5_ = (1 − θ_*nt*_, 1).

Empirical ROC plots have been used in numerous experimental papers as a method for comparing theories, but it is challenging to statistically discriminate between models based on only a few points on the empirical ROC. However, given the historical interest in the ROC in psychology, it is instructive to consider the theoretical ROC for the PD model. See Figure 3 for a general ROC illustration for the PD model. Also see Table 3 for the PD model equations that are linked to key operating points. The table caption describes the definition of the three discrete points illustrated by the open squares in Figure 3, i.e., points *P*_2_, *P*_3_, and *P*_4_. These three points and the two extreme points for the PD model, *P*_1_ and *P*_5_ are a function of the five parameters in the PD model. If 0 < θ_*d*_ < 1, 0 < θ_*nt*_ < 1, and θ_*g*_ > 1 − θ_*g*′_, then the ROC path is along two linear segments. Note that the single-high threshold model discussed by Macmillan and Creelman (2005) is the special case of the PD model when θ_*nt*_ = 0 and θ_*g*_ = 1 − θ_*g*′_. The double-high threshold model also discussed in Macmillan and Creelman (2005) is another special case of the PD model when θ_*nt*_ = θ_*d*_ and θ_*g*_ = 1 − θ_*g*′_.

**Table 3:** **The PD model equations for the key points shown in Figure 3**.

**Point**	***x* = False alarm**	***y* = Hit**
*P*_0_	0	0
*P*_1_	0	θ_*d*_
*P*_2_	(1 − θ_*nt*_)(1 − θ_*g*′_)θ_*h*_	θ_*d*_ + (1 − θ_*d*_)θ_*g*_θ_*h*_
*P*_3_	(1 − θ_*nt*_)(1 − θ_*g*′_)	θ_*d*_ +(1 − θ_*d*_)θ_*g*_
*P*_4_	(1 − θ_*nt*_)(1 − θ_*g*′_θ_*h*_)	θ_*d*_ +(1 − θ_*d*_)(1 − (1 − θ_*g*_)θ_*h*_)
*P*_5_	1 − θ_*nt*_	1
*P*_6_	1	1

*Point P_2_ corresponds to the case where a positive response is considered as a high confident yes, but for point P_3_ a positive is regarded as any yes response. For point P_4_ a positive is considered as any response that is not a high confident no*.

To better understand the PD ROC, consider points *P*_2_ and *P*_3_. If we were to define an affirmative response as strictly a “yes” with high confidence, then the corresponding false alarm rate and hit rate would be illustrated by *P*_2_ and have the values corresponding to the prediction equation shown in Table 3 for that point. Next we redefine an affirmative response as any “yes” response, then the false alarm rate and hit would be illustrated by *P*_3_ and the corresponding prediction equation in Table 3. The slope between *P*_2_ and *P*_3_ is denoted as *s*_23_ and is given as

(1) $$s_{23}=\frac{(1-\theta_{d})\theta_{g}}{\left(1-\theta_{nt})(1-\theta_{g^{\prime }}\right)},$$

and the slope between points *P*_1_ and *P*_2_ is also equal to *s*_23_. The linear path from points *P*_1_ and *P*_3_ can be described in terms of a hypothetical variable *v* that varies on the [0, 1] interval. The false alarm rate *x* and hit rate *y* on this path is described by the following equations:

(2) $$x=(1-\theta_{nt})(1-\theta_{g^{\prime }})v,$$

(3) $$y=\theta_{d}+(1-\theta_{d})\theta_{g}v.$$

The least risky point *P*_1_ corresponds to when *v* = 0. Point *P*_2_ corresponds to the more risky case when *v* = θ_*h*_. Point *P*_3_ corresponds to the even more risky case of *v* = 1. Of course the only observable points on this path from *P*_1_ to *P*_3_ are *P*_2_ and *P*_3_. Interestingly the slope from *P*_3_ to *P*_4_ is in general different than the slope from *P*_1_ to *P*_3_. Let us denote the slope from *P*_3_ to *P*_4_ as *s*_34_, and it is given as

(4) $$s_{34}=\frac{\left(1-\theta_{d})(1-\theta_{g}\right)}{(1-\theta_{nt})\theta_{g^{\prime }}}.$$

It is also the case that the slope from *P*_4_ to *P*_5_ is also equal to *s*_34_. Moreover, the linear path from *P*_3_ to *P*_5_ can be described in terms of another hypothetical variable *w* that varies from 0 to 1 as the risk increases. The false alarms *x* and hits *y* on this path is characterized by the following equations:

(5) $$x=(1-\theta_{nt})(1-\theta_{g^{\prime }}+\theta_{g^{\prime }}w),$$

(6) $$y=\theta_{d}+(1-\theta_{d})\theta_{g}+(1-\theta_{d})(1-\theta_{g})w.$$

The *P*_3_ point corresponds to *w* = 0; whereas the *P*_4_ point corresponds to *w* = 1−θ_*h*_ and *P*_5_ corresponds to *w* = 1.

**Figure 4:**
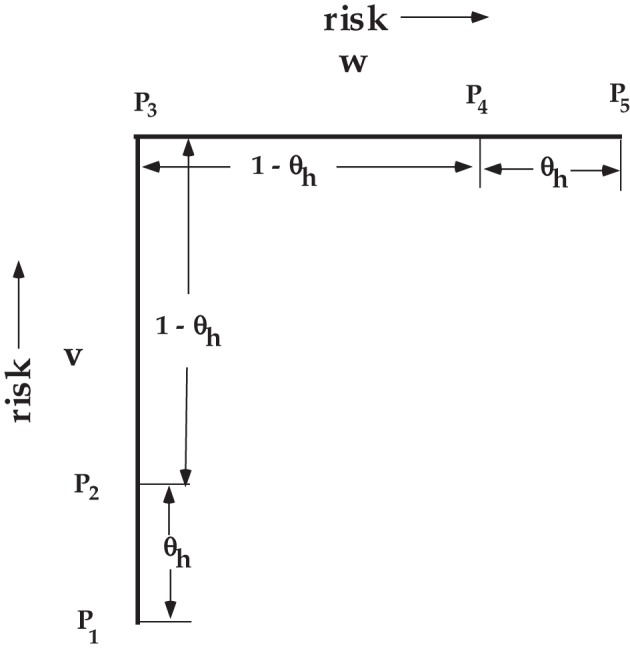
**Illustration of the relative position of the *v* and *w* variables that determine the points on the PD ROC**. See Figure 3 for the definition of the points.

Figure 4 illustrates the PD model ROC path from one extreme point to the other in terms of the *v* and *w* variables. As *v* varies from 0 to 1 it traces points on the *P*_1_ to *P*_3_ line as stipulated by Equations (2, 3). Similarly as *w* varies from 0 to 1, (Equation 5) and (Equation 6) traces points on the *P*_3_ to *P*_5_ line. Notice that θ_*h*_ determines the separation from each of the two extreme ends. This feature is a property of the PD model because there is a common parameter of incorrectly using the high confidence rating when guessing regardless if the guessing is done in either the target-present condition or the target-absent condition. Chechile (2004) also presented another identifiable memory MPT model where there are separate parameters for over confidence when using the “yes” response (θ_2_) versus over confidence when using the “no” response (θ_1_). This model is the 7B model. Other than the difference in the handling of over confidence, the 7B and 6P models are identical, i.e., the 6P model is the special case of 7B where θ_*h*_ = θ_1_ = θ_2_. Model 7B can also be applied to the perceptual detection task (lets denote that model as the PD^*^ model). In the PD^*^ model the θ_2_ parameter determines the location for the *v* variable for the *P*_2_ point, and the θ_1_ parameter determines the separation for the *w* variable from the maximum of 1. Hence, the spacing for the points on the *v* − *w* plot is different for the PD^*^ model than the spacing shown in Figure 4 for the PD model.

In general the slope from *P*_3_ to *P*_5_ is less than the slope from *P*_1_ to *P*_3_. Given Equations (1), and (4) the ratio of the slopes can be written as

(7) $$r=\frac{s_{35}}{s_{13}}=\frac{\left(1-\theta_{g})(1-\theta_{g^{\prime }}\right)}{\theta_{g}\theta_{g^{\prime }}}.$$

If there is some partial or degraded perception, then the tendency to respond “yes” is at least equal or greater in the target-present condition as it is in the target-absent condition. It follows that

(8) $$\frac{\theta_{g}}{1-\theta_{g}}\geq \frac{1-\theta_{g^{\prime }}}{\theta_{g^{\prime }}}.$$

It also follows from Equations (7, 8) that *r* ≤ 1. Consequently, if θ_*g*_ > 1 − θ_*g*′_, then the slope from *P*_1_ to *P*_3_ is larger than the slope from *P*_3_ to *P*_5_. The case where *r* = 1 corresponds to when θ_*g*_ = 1 − θ_*g*′_ or when there is the same “yes” guessing in the target-present condition as in the target-absent condition. In this special case, there is no partial detection, and the ROC does not have two linear components, but there is instead a single line of slope $\frac{1-\theta_{d}}{1-\theta_{nt}}$ between *P*_1_ and *P*_5_.

The area under the ROC has been used as a measure of sensitivity in standard SDT. It is straightforward to show that area *A*_*c*_ between the *P*_1_ − *P*_5_ dashed line in Figure 3 and the main diagonal line of *y* = *x* is $\frac{1}{2}$ (θ_*d*_ + θ_*nt*_ − θ_*d*_ θ_*nt*_)^8^. This region is a function of certain perceptual detection and does not depend on guessing. Because the total area in the upper half of the unit square where *y* > *x* is $\frac{1}{2}$, it is advantageous to multiply *A*_*c*_ by 2, so that the area measure of certain detection is placed on a 0 to 1 scale. This measure is defined as a certain detection *D*_*c*_, and

(9) $$D_{c}=\theta_{d}+\theta_{nt}-\theta_{d}\theta_{nt}.$$

The area of the *P*_1_ *P*_3_ *P*_5_ triangle is a function of guessing. This area is denoted as *A*_*g*_, and it can be found from Heron's formula, i.e., *A*_*g*_ = $\frac{1}{2}$(1 − θ_*nt*_)(1 − θ_*d*_)[θ_*g*_ − (1 −θ_*g*′_)]. We can put this measure of effective guessing on a 0 to 1 scale by defining *D*_*g*_ = 2*A*_*g*_ or

(10) $$D_{g}=(1-\theta_{nt})(1-\theta_{d})[\theta_{g}-(1-\theta_{g^{\prime }})].$$

Thus the total detection measure can be defined as twice the area between the ROC and the main diagonal; this metric is *D* = *D*_*c*_ + *D*_*g*_ or

(11) $$D=\theta_{d}+\theta_{nt}-\theta_{d}\theta_{nt}+(1-\theta_{nt})(1-\theta_{d})[\theta_{g}-(1-\theta_{g^{\prime }})],$$

As an example, let us compute these area-based metrics for the radiological data discussed in section **2.2**. Using PPM estimates for θ_*d*_ and θ_*nt*_, it follows from Equation (9) that *D*_*c*_ = 0.774. The corresponding *D*_*g*_ measure from Equation (10) is 0.031, so the overall *D* metric is 0.805.

Although the detection measure *D* is on a proportional basis, it is, nonetheless, a confounded measure because it does not delineate how the detection was achieved. For example suppose that θ_*nt*_ = 0.805 and θ_*d*_ = 0, then the resulting *D* value would be the same as for the radiologist discussed above. Clearly the hypothetical observer with θ_*d*_ = 0 and θ_*nt*_ = 0.805 would be very good at recognizing a normal CT scan, but would not be capable of detecting an abnormal scan, which would be a rather serious problem for the diseased patients of that hypothetical radiologist! Consequently, the area-based *D* metric, along with its component metrics of *D*_*c*_ and *D*_*g*_, is less informative as the original PD model parameters. The detection of the target increases with the value of the θ_*d*_ parameter, and the identification of a non-target increases with the value of the θ_*nt*_ parameter. Those two types of detection can be quite different. It is also informative to know how the observer does for the unclear cases where there is guessing. The *D* metric does not pull out the many different perceptual and decision-making characteristics of the observer's behavior. Also the standard SDT metrics of *d*′ and the ratio of the standard deviations do not extract the different properties of the observer's perceptual-detection performance.

## 3. Individual difference estimation for the PD model

A fundamental issue that arises in mathematical psychology is the basis for fitting a model. One method is to fit the model separately for each individual and to average individual estimates for the group average. Another method is to aggregate the data across a group of individuals for a particular experimental condition and then fit the model once for that condition^9^. The estimates from these two approaches differ. Although there are applications where each of these pure approaches is reasonable, in this paper a hybrid of these two methods will be recommended. Consequently, the answer to the question as to how to fit a model depends on the purpose of the analysis.

There are several contexts that necessitate the fitting of the model on an individual basis. For example, if the model is a non-linear function of an independent variable, then many investigators have demonstrated that group-averaged data can result in biased fits (Estes, 1956; Sigler, 1987; Ashby et al., 1994). Also the theoretical issue being examined can require that the analysis be done on an individual basis. For example, Chechile (2013) examined the memory hazard function to see if there was evidence of a mixture over stimuli. Had that analysis been done on a grouped-data basis, then any results suggesting a mixture could have been a mixture over individuals with different memory properties instead of a mixture over stimuli.

There are also cases when pooling the data prior to the model fit is the preferred analysis (Cohen et al., 2008; Chechile, 2009). Chechile (2009), for example, studied four prototypic MPT models with an extensive series of Monte Carlo simulations in order to examine the relative accuracy of averaging versus data pooling. For any given Monte Carlo run, a group of *n*_*g*_ simulated “subjects” with slightly different true values for the model parameters was constructed, and for each artificial subject there were *n*_*r*_ “observations” that were randomly sampled from the appropriate multinomial likelihood distribution^10^. Based on this set of simulated outcome frequencies, the model was fit in two different ways: (1) the averaging method and (2) the data-pooling method. For the averaging method the MPT model was fit separately for each of the *n*_*g*_ subjects, and these estimates were averaged to obtain an estimate for each model parameter. For an arbitrary model parameter, θ_*x*_, the group average estimate is ${\bar{\theta }}_{x}=\frac{1}{n_{g}}\sum_{i=1}^{n_{g}}{\hat{\theta }}_{xi}$ where $\hat{\theta }$_*x i*_ is the parameter estimate for the *i*th subject. For any Monte Carlo run, the absolute value difference was computed between θ_*x*_ and the true mean for that parameter $\theta_{x}(true)=\frac{1}{n_{g}}\sum_{i=1}^{n_{g}}\theta_{xi}(true)$. This difference is taken as the error for the averaging method for that one Monte Carlo run. The process was then repeated so that in total there were 1000 separate Monte Carlo runs for each combination of *n*_*g*_ and *n*_*r*_. Across these separate Monte Carlo runs the model parameters were varied, so the model was simulated over a vast set of configurations of the parameters. The overall error for the averaging method is the mean error across the 1000 Monte Carlo data sets for each combination of *n*_*g*_ and *n*_*r*_. For the identical data as described above, a corresponding error was also found for the pooling method. For the pooling method the frequencies in each multinomial response category was summed across the *n*_*g*_ subjects in a group, and the model was fit once with the pooled data. The estimate based on pooling for the *j*th simulated data set is denoted as $\hat{\theta }$_*x j*_(*pooled*). The absolute value difference between this estimate and the true value for that run is the pooling error for the *j*th Monte Carlo data set, and mean error across all 1000 data sets is the overall error for the pooling method^11^. For all four models reported in Chechile (2009) and for most combinations of *n*_*g*_ and *n*_*r*_, the mean error for the pooling method was less than the corresponding error obtained for the averaging method^12^. Consequently, Chechile (2009) reported a pooling advantage score that was the difference between the mean averaging error and the mean pooling error. For example, a positive value for the pooling advantage score of 0.07 means that the averaging mean error was larger by 0.07 than the corresponding pooling error. A negative pooling advantage score would mean that the averaging method had less error than the pooling method.

One of the models examined in Chechile (2009) was a four-cell MPT model that is identical to the structure of the process trees for either the target-present or the target-absent test conditions with the PD model. Consequently, those Monte Carlo simulations directly apply to the PD model. Table 4 provides a condensed summary of the Monte Carlo results from Chechile (2009). The θ_*d*_ parameter in Table 4 corresponds to the θ_*S*_ parameter in Model A; whereas θ_*g*_ and θ_*h*_, respectively, correspond to the θ_*g*_ and θ_1_ parameters in Model A.

**Table 4:** **The difference in mean error between averaging and pooling for *n_g_* individuals in a group and for *n_r_* trials in the target-present condition**.

	**Pooling Advan. score**		
***n_g_ n_r_***	***θ_d_***	***θ_g_***	***θ_h_***
20 20	0.069	0.076	0.078
20 50	0.045	0.050	0.051
20 100	0.034	0.035	0.034
20 400	0.015	0.013	0.013
40 20	0.078	0.086	0.087
40 50	0.054	0.059	0.059
40 100	0.037	0.040	0.040
40 400	0.017	0.015	0.015
80 20	0.087	0.096	0.098
80 50	0.059	0.065	0.064
80 100	0.043	0.043	0.045
80 400	0.020	0.016	0.016

*This difference is a pooling advantage score. Positive values indicate less error for the pooling method. Monte Carlo simulations from Chechile (2009)*.

The pooling advantage scores in Table 4 exhibit a number of interesting properties that were also found with the other MPT models. First, the pooling advantage scores are positive indicating that there is greater accuracy for the pooling method. Second, although the magnitude of the pooling advantage decreases with the number of observations per subject (*n*_*r*_), there is still a non-trivial advantage for pooling even when *n*_*r*_ = 400. It is challenging to do an experiment with large values for *n*_*r*_. For example, a replication number of 50 is larger than all but two of the memory studies reported from my laboratory. Consequently, the idea of running a large number of replication trials per subject is not a practical option. Third, the size of the pooling advantage increases with group size *n*_*g*_. This effect is due to the fact that the error for the pooling method decreases rapidly with increasing group size; whereas the error for the averaging method slowly decreases with increasing *n*_*g*_, so the net effect is that the pooling advantage score increases with *n*_*g*_.

It might not seem intuitive as to why the pooling of data results in superior estimates for the group mean. This result is more reasonable when viewed from a Bayesian perspective. From Bayes theorem it does not matter if the data are examined in aggregate or one observation at a time, provided that the same starting prior probability is used. Suppose we use a uniform distribution as the prior distribution for each combination of the parameters (θ_*d*_, θ_*g*_, θ_*h*_). Let us call this prior the “vague” prior. Furthermore suppose we examine the model parameters for the first individual in the group via Bayes theorem to yield a posterior distribution. The posterior distribution after the first individual should then be the prior distribution for examining the data for the second subject, i.e., it is no longer appropriate to maintain the vague prior after examining the first subject. Similarly the prior distribution for Subject 3 should be the posterior distribution after considering the first two subjects. This one-subject-at-a-time method eventually yields a posterior distribution that is the same as the posterior distribution achieved by pooling the multinomial categories and applying Bayes theorem once. Had the Bayesian analyst used a vague prior for each of the *n*_*g*_ subjects and averaged the estimates, then the analysis would not be consistent in the application of Bayes theorem. The averaging of separate estimates is not an operation by which probability distributions are revised via Bayes theorem. In terms of this framework, the findings in Table 4 are quite reasonable. The pooling method should be more accurate, and the pooling advantage should grow with the size of the group.

Despite the above demonstration of a pooling advantage for estimating the group mean, it is still an open question as to what should be the basis for estimating the model parameters for an individual. Two choices seem reasonable. One method is simply to use the data for just the individual, e.g., for the θ_*d*_ parameter it would be $\hat{\theta }$_*d i*_ for the *i*th observer. For the second method the data for the individual is used but there is a fixed correction so that the mean across all observers is equal to the pooled estimate for the group. For the θ_*d*_ parameter this estimate is denoted as $\hat{\theta }$^(*a*)^_*d i*_ and is defined as

(12) $${\hat{\theta }}_{di}^{\left(a\right)}={\hat{\theta }}_{d}(pooled)-{\bar{\theta }}_{d}+{\hat{\theta }}_{di}.$$

Note that the two methods have estimates that are perfectly correlated because the adjusted estimate $\hat{\theta }$^(*a*)^_*d i*_ is a constant plus the individual estimate $\hat{\theta }$_*d i*_. The constant correction term is equal to $\hat{\theta }$_*d*_(*pooled*) − θ_*d*_. The correction makes the mean of the adjusted estimates equal to the pooling method estimate because

$$\frac{1}{n_{g}}\sum_{i=1}^{n_{g}}{\hat{\theta }}_{di}^{\left(a\right)}={\hat{\theta }}_{d}(pooled)-{\bar{\theta }}_{d}+{\bar{\theta }}_{d}={\hat{\theta }}_{d}(pooled).$$

The estimate based on Equation (12) is similar in principle to a James–Stein estimator used for the linear model for Gaussian random variables because the estimate for the individual is shifted based on properties of the group.

Another Monte Carlo simulation was designed for a widely different group of simulated observers in order to assess the relative accuracy of the two methods for estimating the parameters for individuals. The group consisted of 10 observers for each of the 3 × 3 combinations of values for θ_*d*_ and θ_*nt*_. The three values were 0.2, 0.5, and 0.8. For each of the 90 simulated observers the values for θ_*h*_ were randomly selected from a beta distribution with coefficients of 2 and 4, and the θ_*g*_ and θ_*g*′_ parameters were randomly selected from a beta distribution with coefficients of 28 and 14. Consequently true scores were established for each simulated observer. For each observer, 20 simulated observations were randomly sampled for the target-present condition, and another 20 observations were randomly sampled for the target-absent condition. These observations were based on the appropriate multinomial likelihood distribution for each subject. The PD model was then estimated by each method described above. Because θ_*d*_ and θ_*nt*_ are the two key parameters of interest in the PD model, the root mean square (rms) error was found between the true score point {θ_*d i*_(*true*), θ_*nt i*_(*true*)} and the estimated point for the individual {$\hat{\theta }$_*d i*_, $\hat{\theta }$_*nt i*_}. The rms error for the adjusted score point {$\hat{\theta }$^(*a*)^_*d i*_, $\hat{\theta }$^(*a*)^_*nt i*_} was also found. The rms errors for the individual and the adjusted method are respectively 0.1671 and 0.1385. Thus, the adjusted estimates based on Equation (12) resulted in a 17% reduction in the rms error. This simulation illustrates the improvement in the accuracy of model estimation by the use of the adjusted score method.

## 4. Discussion

In this paper the Chechile (2004) 6P memory measurement model was modified and applied to perceptual detection. The resulting PD model is a MPT model that has two mixture rate parameters (θ_*d*_ and θ_*nt*_) that measure the proportion of times that the observer confidently detects something that belongs to an identifiable category. The categories are different for targets and non-targets, but in both cases something is being identified. The measurement of these detection rates is an important part of the psychometric assessment of perceptual performance. The PD model also has three other parameters that come into play when the observer is unable to confidently classify the stimulus.

The PD model differs from standard SDT on the issue of stochastic mixtures. MPT models, like the PD model, are essentially probability mixture models. In contrast, SDT developed in the context of assuming separate but homogeneous distributions for target-present and target-absent conditions. The success of the PD model in accounting for the radiological judgments described earlier in this paper occurred because the PD model was sensitive to the fact the radiologist was able to know sometimes that a CT scan was normal and to know at other times that a CT scan revealed an identifiable abnormality. This attribute of categorical and sophisticated perception is not an isolated property of experts. More than 120 years ago William James discussed the importance of perceptual learning; in fact perception according to James differed from a pure sensation because of the information that the person associates and adds to the sensation (James, 1890). There is now a vast literature describing the improvement in perception with practice (Kellman, 2002). With experience people can develop refined perceptual categories that sharpen their ability to process and to interpret stimuli.

It is noteworthy that the prototypic experiments in the early history of SDT used stimuli that were designed to be featureless and varied on only a single prothetic intensity dimension. For example the stimulus-absent stimulus for some experiments was white noise; whereas the target-present stimulus was a louder white noise (Tanner et al., 1956). Perceptual categories and perceptual learning is limited for such impoverished stimuli. SDT is expected to be quite successful for such applications, but SDT is expected to be problematic when stimuli possess rich perceptual features and when the observer has some experience with the class of stimuli. For those applications, the PD model would be a more suitable cognitive psychometric tool for assessing the properties of the observer.

The PD model is a minimalistic model that intentionally eschews delineating any specific cognitive representation of the stimulus. Like other MPT models, there are probability measures for specific states. The states for the PD model are: (1) a state of certain target recognition, which occurs on θ_*d*_ proportion of the target-present trials, and (2) the state of certain identification of something other than a target, which occurs on θ_*nt*_ proportion of the target-absent trials. These probability measures provide for a characterization of the observer's detection ability.

MPT models have many desirable statistical properties and can be estimated by a variety of methods. Monte Carlo simulations with large sample sizes demonstrated that the MLE and the Bayesian posterior mean for the PD model were very close, but the accuracy of these estimates differed more substantially for smaller sample sizes. When the estimates differ, the Bayesian mean was found to be more accurate. In addition, an improved estimate was found for the individual observer when the estimate based on the individual's data was adjusted. The adjustment was a fixed amount for all observers, and it equated the mean of the adjusted scores to the mean of the estimate based on pooled data. This adjustment was discussed as an analogous adjustment to the James–Stein shrinkage improvements to the MLE found for the multiple-group Gaussian model.

### Conflict of interest statement

The author declares that the research was conducted in the absence of any commercial or financial relationships that could be construed as a potential conflict of interest.

## Footnotes

^1^Some MPT models have been characterized as threshold models by the authors of the model (e.g., the two high-threshold model of Snodgrass and Corwin, 1988). A threshold is an activation level on an underlying strength continuum that triggers the memory to be in a given state. The assumption of thresholds in MPT models has been vigorously challenged by researchers who prefer a SDT perspective (viz. Dube and Rotello, 2012). However, the concept of a mixture over different knowledge states does not require the assumption of a threshold. For example in the Chechile (2004) 6P model, the knowledge states discussed above are not driven by an underlying strength, but rather it is based simply on the existence or not of specific memory content.

^2^See Chechile (1977, 1998, 2004) for a more detailed discussion of model identifiability.

^3^The MCMC method is an implementation of the Metropolis–Hastings algorithm after an initial “burn in” period of 300,000 cycles for sampling each model parameter.

^4^There is a difference in the prior distributions used for the MCMC method and for the PPM method. For the MCMC approach, a flat prior is assumed for each of the PD model parameters, i.e., the (θ_*d*_, θ_*nt*_, θ_*g*_, θ_*g*′_, θ_*h*_) parameters. However, for the PPM method the prior is a flat distribution for the multinomial cell proportions shown in Figure 1, i.e., the (ϕ_*i*_) parameters. The joint posterior distribution for the (ϕ_*i*_) parameters is a product of two Dirichlet distributions. With the PPM method, random samples of (ϕ_*i*_) values are taken from the posterior distribution, and each vector of (ϕ_*i*_) values is mapped to a corresponding vector of the PD model parameters.

^5^There were six cases for the normals where the radiologist used another category called questionable. Three of these cases are assigned here to the second category (probably normal), and three cases were assigned here to the third category (probably abnormal).

^6^There were two CT scans for the abnormals that the radiologist gave the response of questionable. One of these cases was assigned here to the second category, and one was assigned here to the third response category.

^7^Given the values for *p*_1_ = ϕ_1_, *p*_2_ = ϕ_1_ + ϕ_2_, and *p*_3_ = ϕ_1_ + ϕ_2_ + ϕ_3_ there are three decision points for randomly assigning a simulated “observation” to one of the four cells. For each simulated observation, a random score is sampled from a uniform distribution on the (0, 1) interval. If the random score is less than *p*_1_, then the observation is for cell 1. If the random score is in the [*p*_1_, *p*_2_) interval, then it is an observation for cell 2. If the random score is in the [*p*_2_, *p*_3_) interval, then the observation is for cell 3. If the random score is greater or equal to *p*_3_, then it is an observation in cell 4.

^8^Note that the total area above the main diagonal is $\frac{1}{2}$, and the area above the dashed line is $\frac{1}{2}$(1 − θ_*d*_)(1 − θ_*nt*_), so *A*_*c*_ can be determined by subtracting these quantities.

^9^A third approach also exists for obtaining individual and group effects by means of a hierarchical Bayesian model similar to the analysis developed for MPT models by Klauer (2010). This method is computationally challenging, and it has not yet been assessed to see if it has improved accuracy relative to the simple model advanced in the present paper.

^10^Each individual was within ±0.03 of the group mean.

^11^This whole procedure of estimating the model with both the averaging and pooling method was done for both PPM and MLE estimates for each of the four typical MPT models.

^12^Only eight cases out of 640 cases reported in Chechile (2009) had greater error for the pooling method, and all of these exceptions were when the MLE was used. Generally the MLE was not the optimal estimator for the model parameters because the corresponding Bayesian PPM estimator had greater accuracy.
